# What Enables Size-Selective Trophy Hunting of Wildlife?

**DOI:** 10.1371/journal.pone.0103487

**Published:** 2014-08-06

**Authors:** Chris T. Darimont, K. Rosie Child

**Affiliations:** 1 Department of Geography, University of Victoria, Victoria, BC, Canada; 2 Raincoast Conservation Foundation, Sidney, BC, Canada; University of California, Berkeley, United States of America

## Abstract

Although rarely considered predators, wildlife hunters can function as important ecological and evolutionary agents. In part, their influence relates to targeting of large reproductive adults within prey populations. Despite known impacts of size-selective harvests, however, we know little about what enables hunters to kill these older, rarer, and presumably more wary individuals. In other mammalian predators, predatory performance varies with knowledge and physical condition, which accumulates and declines, respectively, with age. Moreover, some species evolved camouflage as a physical trait to aid in predatory performance. In this work, we tested whether knowledge-based faculty (use of a hunting guide with accumulated experience in specific areas), physical traits (relative body mass [RBM] and camouflage clothing), and age can predict predatory performance. We measured performance as do many hunters: size of killed cervid prey, using the number of antler tines as a proxy. Examining ∼4300 online photographs of hunters posing with carcasses, we found that only the presence of guides increased the odds of killing larger prey. Accounting for this effect, modest evidence suggested that unguided hunters presumably handicapped with the highest RBM actually had greater odds of killing large prey. There was no association with hunter age, perhaps because of our coarse measure (presence of grey hair) and the performance trade-offs between knowledge accumulation and physical deterioration with age. Despite its prevalence among sampled hunters (80%), camouflage had no influence on size of killed prey. Should these patterns be representative of other areas and prey, and our interpretations correct, evolutionarily-enlightened harvest management might benefit from regulatory scrutiny on guided hunting. More broadly, we suggest that by being nutritionally and demographically de-coupled from prey and aided by efficient killing technology and road access, wildlife hunters in the developed world might have overcome many of the physical, but not knowledge-based, challenges of hunting.

## Introduction

The requirements to detect, pursue and capture prey have in part shaped the evolution of mental and physical faculties among all mammalian predators. For example, detection requires a capacity for knowledge so targeted prey can be reliably located and effectively subjugated [1], [2]. To avoid early detection themselves, some predators evolved physical camouflage [3]–[7]. During pursuit and capture stages, traits related to physical fitness are also important. Stalking and ambushing predators, for example, need the ability to accelerate, while coursing predators require stamina [2], [8], [9].

As predators age, individuals gain relevant knowledge but also, after a certain time, accumulate physical handicaps. In carnivores, for example, predatory success (defined as kill rate) increases with age (and associated experience), even years after adult body size is reached (*e.g.,* [2], [10], [11]). However, at more advanced ages predatory senescence (declining predatory success) has recently been documented in mammals and birds [2],[12],[13].

Contemporary wildlife hunters function fundamentally as predators, and can impose remarkable ecological and evolutionary influence. Festa-Bianchet (2003), for example, estimated that hunters are responsible for more predation on adult wildlife in North America than carnivores ([14], *see also* [15]). Moreover, emerging evidence suggests that hunter preference for large, reproductive-aged males can influence not only age, sex, and social structures within populations [16], but also the selective landscape for morphological, life history, and behavioural traits [14], [17]–[19]. Responses in ungulate populations can include declines in body or ornament size following the targeted removal of larger individuals (*e.g.,* [20], [21], *but see* [22]). Whereas work that measures responses by prey to size-selective harvests are now common, only rarely does research examine the predator common among studies.

Like other predators, wildlife hunters vary individually in knowledge-based and physical traits and – although to our knowledge unexplored – these characteristics might also govern predatory performance. Whereas ‘human predators’ might possess advanced intellectual abilities compared to other mammals, they are comparatively weak, awkward, minimally camouflaged, and lack natural weapons such as claws or fangs [23]. On the other hand, manufactured weapons reduce the influence of these handicaps; from early spears to long-distance projectile weaponry that allowed killing at a distance, weapon technology has evolved to allow hunters to kill large and fast animals [23], [24]. For contemporary wildlife hunters in developed regions, vehicles, extensive road systems, optics, and bullets can increase efficiency and minimize costs of detection and, in many cases, obviate a pursuit phase. Additionally, many hunters can instantaneously adopt camouflage; such clothing is commonly worn under the assumption that it reduces the probability of detection by prey. Finally, some hunters employ guides, who serve as specialized hunters that offer local knowledge and assistance, presumably improving outcomes of hunts. Guides can additionally grant access to hunters into lightly-hunted areas. Among Alaskan moose hunters, for example, Schmidt et al. [25] found that guided hunters killed larger moose (*Alces alces*) than unguided hunters, likely because guides provide hunters knowledge of (and access to) low-density areas that produced large-antlered moose (*but see* [26]).

Despite these advantages, additional handicaps to physical performance are presumably pronounced among contemporary wildlife hunters. Chief among them is that humans are now among the fattest of all mammals [27], [28]. If hunting requires some measure of physical fitness, and the abilities of wildlife hunters are compromised by extra body mass, one might expect hunting performance to be reduced for those with the greatest relative body mass (hereafter RBM). On the other hand, the interaction between hunting technology (i.e., long-range rifles) and landscapes (with easy road and vehicle access to wildlife habitat) in which much hunting now occurs might have minimized the physical demands to the point that poor physical fitness no longer poses a handicap. Advanced age might also negatively affect hunt performance. Although older hunters might have accrued more knowledge, they might be handicapped by poorer physical condition that accompanies aging.

We used relative size (i.e., number of antler tines) of killed cervids (elk [*Cervus canadensis*], mule deer [*Odocoileus hemionus*], white-tailed deer [*Odocoileus virginianus*]) as a proxy for hunting performance to examine if and how mental and physical traits as well as age might be important in wildlife hunting. We used prey size as our measure of hunting performance for three reasons. First, larger specimens are typically older, rarer, and often more vigilant individuals within populations [29]–[31], thereby posing greater challenges to hunters. Second, larger individuals are those targeted and valued by many hunters [14]. Finally, understanding what traits might enable size-selective harvests might provide utility to wildlife management; such size-selective hunting behaviour can in principle invoke undesirable phenotypic responses in cervid prey, namely in reduced body or ornament size (above; [14], [17], [20], *but see* [22]).

Using data from online hunting photographs, which provide information about hunters and their killed prey, we tested whether knowledge-based faculty (use of a hunting guide), physical characters (RBM and camouflage clothing), and age predicted the relative size of killed prey. We made naïve predictions that guides, low RBM, and camouflage might increase the odds that hunters could kill large specimens. Given how mental and physical performance might vary with age, we could not predict whether and how the odds of securing large game might vary with hunter age.

## Methods

**Figure 1:**
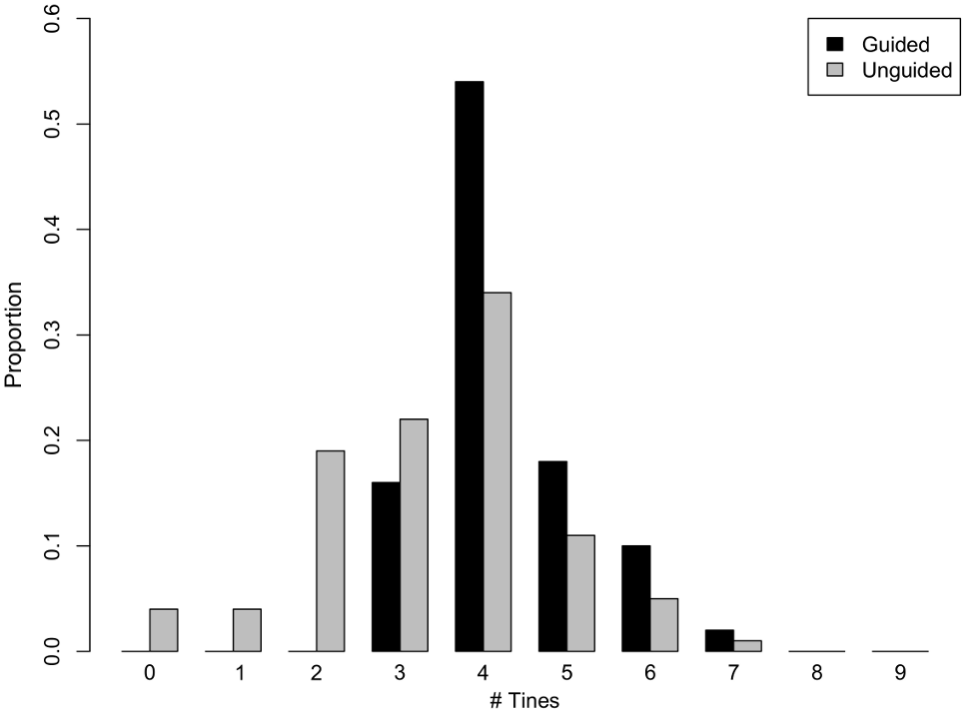
Distribution of tine numbers for guided (black) and unguided (gray) hunters across the three species.

**Figure 2:**
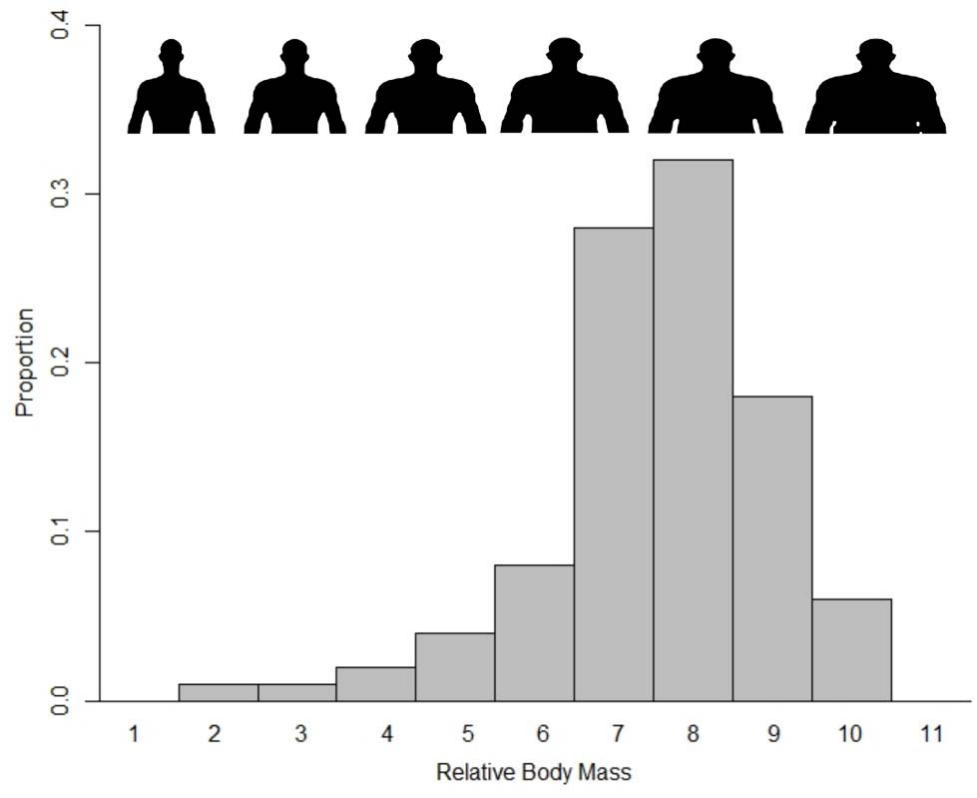
Distribution of relative body mass (RBM) of hunters from British Columbia and Alberta, Canada, sampled from social media data. Visual anchors left to right at 1, 3, 5, 7, 9 and 11. To protect the privacy of hunters in online photographs, we used cartoon images of men with appropriate morphology to display variation in RBM.

We obtained 5,202 photographs of adult (>18 years) male hunters in British Columbia (BC) and Alberta, Canada, posing alone with cervid prey. Images were downloaded from professional guide outfitter websites (guided; n = 3666) and online hunting forums (unguided; n = 629). We classified prey as small or large, with individuals possessing more than its species mode tine number as large (n = 807) and others as small (n = 3488). The threshold for large and small specimens was six for elk and four for both species of deer. The distribution of tine numbers for guided and unguided hunters across the three species is shown in Figure 1. We coarsely classified hunters as young (n = 2770) or old (n = 1525), based on presence of grey or white hair. One person (KRC) scored each hunter’s RBM on an 11 point scale, based on deposits of adipose tissue in the face, neck, and when visible, abdomen; this method reliably predicts health and mortality in photographs of sampled individuals [32]. Scores varied among hunters with modal values ranging from 7–9 (Figure 2). Data from global assessments of obesity reveal that approximately 23% of Canadians, 33% of Americans and 11–23% of Europeans are obese (Body Mass Index [kg/m^2^] ≥30; [28]). Assuming hunters comprise a representative profile of the general population, we classified the 24% of hunters with the highest RBM (categories 9–11; n = 1037) as large and the remainder (n = 3258) as small. Finally we scored the presence (n = 3419; 80% of hunters) or absence of any visible camouflage clothing among hunters posing with killed prey.

Photographs in which any variable could not be assessed were disqualified (n = 907), leaving 4,295 potentially usable cases. Precision for hunter age was previously assessed for these photographs (95% overall proportional agreement; Child & Darimont, unpublished data). To evaluate precision for RBM, we first assigned a confidence rating to each photo for this variable (high, medium, low). Once all pictures were scored, a third party presented KRC with a 6% random subset of images (n = 260) to re-score. Proportional agreement (small vs. large categories) was 87%. Treating RBM as a continuous variable (i.e., raw values from 1–11) reduced proportional agreement to 52%, which led us to categorize this measure.

We used an information theoretic approach to rank candidate models that had a generalized linear form (GLM). We specified a binomial link to estimate the potential effects of hunter age, RBM, guide assistance, and camouflage clothing on prey size. Candidate models consisted of combinations of variables (and some interactions among them) we predicted *a priori* would reasonably explain hunting success (Table S1 in File S1). We inspected the data for model fit and found no evidence of over dispersion (Hosmer-Lemeshow GOF statistic = 5.49, df = 8, p = 0.70). We used Akaike’s Information Criterion (AIC) to guide top model selection and report model-averaged parameter estimates and odds ratios [33]. In separate models otherwise identical in form, we treated RBM as a continuous variable (as opposed to categorical); results were similar and are reported in Tables S2 & S3 in File S1.

### Ethics Statement

All protocols followed in this study were carried out in accordance with the recommendations of the Human Research Ethics Board of the University of Victoria. The human protocol was approved by the University of Victoria Human Research Ethics Board (Protocol Number 13–338). Consent was obtained for the use of these photographs from the University of Victoria Human Research Ethics Board.

## Results

**Figure 3:**
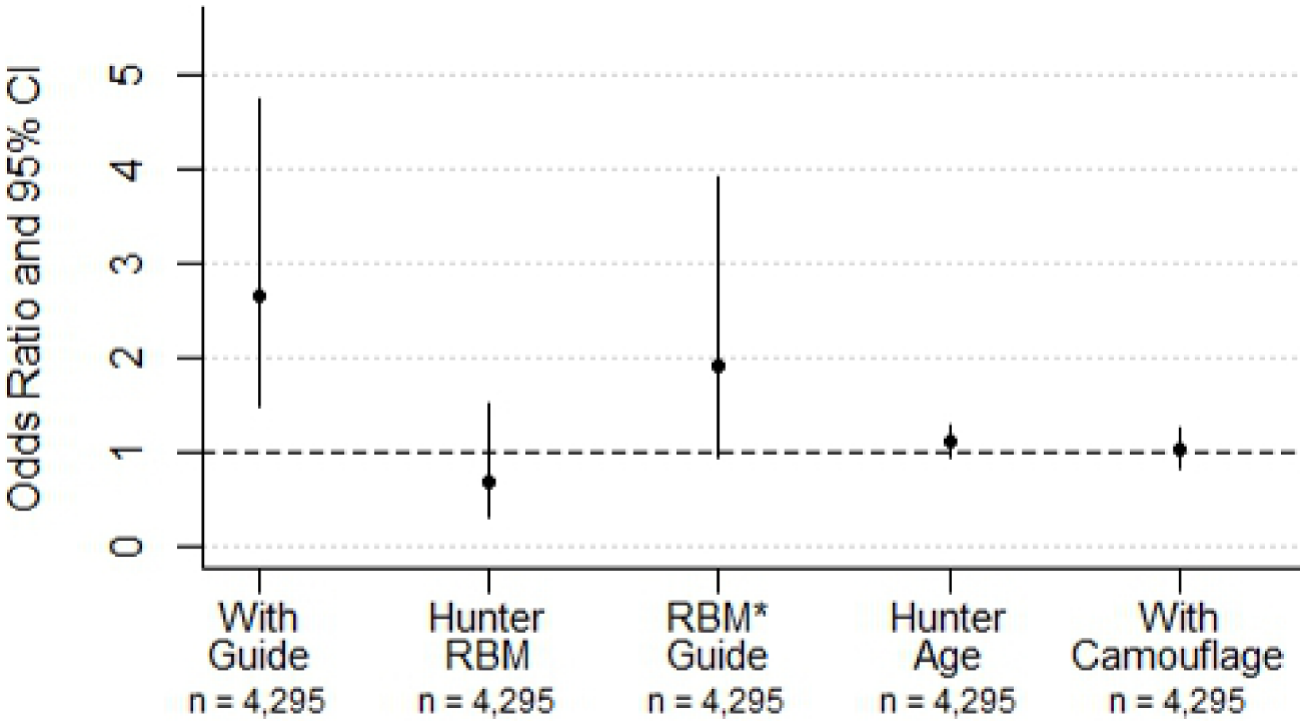
The odds of hunters from British Columbia and Alberta, Canada, displaying a large cervid carcass as a function of the presence of a guide, relative body mass (RBM), age, and camouflage clothing. Among unguided hunters, those with higher RBM had increased odds of killing large prey, though this effect was modest (P = 0.07).

Hunter knowledge, specifically the presence of a guide, was far more important than other hunter characteristic in predicting size of killed prey (Figure 3). Whereas all four variables appear in the top model set (ΔAIC<2; Table 1), inference from model-averaged parameter estimates suggests that only the presence of a guide had a significant effect (Table 2). This variable appeared in all models and was more than twice as important (∑ω = 1) as the next influential variable, RBM (∑ω = 0.45). The odds of guided hunters killing a large cervid were 2.7 times greater than unguided hunters (odds ratio 2.66; 95% CI 1.49–4.75; P<0.01). Alone, hunter body mass had no significant effect (P = 0.35). Among unguided hunters, however, individuals with high RBM actually had increased odds of killing larger prey (interaction term; odds ratio 1.92; 95% CI 0.94–3.92), though this effect was marginal (P = 0.07). Despite 80% of hunters wearing camouflage clothing, this did not affect the odds of securing large game (∑ω = 0.10; odds ratio 1.03; 95% CI 0.84–1.25; P = 0.10). Likewise, the age of hunters was unimportant (∑ω = 0.29; odds ratio 1.10; 95% CI 0.94–1.29; P = 0.23). Although candidate models included interactions among hunter age and RBM as well as hunter age and guide presence, models with these terms did not occur in the top model set.

**Table 1:** Top Models (ΔAIC≤2) to predict the odds of wildlife hunters killing large cervids in British Columbia and Alberta, Canada.

Model Form	ΔAIC	ω_i_
Guide	0.0	0.22
guide, RBM, guide*RBM	0.3	0.19
guide, age	0.5	0.17
guide, RBM	1.3	0.12
guide, camo	1.9	0.09
guide, RBM, age	1.9	0.09

**Table 2:** Model-averaged parameter estimates and relative importance derived from inference across the top model set.

Variable	Estimate	P	Odds Ratio	95% CI	∑AIC ω_i_
Guide	0.98	**<0.01**	2.66	1.49–4.70	1.00
RBM	−0.37	0.35	0.69	0.31–1.51	0.45
Age	0.10	0.23	1.10	0.94–1.29	0.29
guide*RBM	0.65	**0.07**	1.92	0.94–3.92	0.22
camo	0.02	0.80	1.03	0.84–1.25	0.10

P values<0.10 bolded.

## Discussion

We harnessed a relatively new data source (social media) to explore what characteristics among contemporary wildlife hunters might enable size-selective harvests, one measure of hunting performance. Although patterns emerged, we acknowledge some limitations of our approach. For example, we counted antler tines as a proxy for prey size. This restricted our survey to antlered species and provides only one measure of intra-population variation in size, age, and potential wariness/difficulty of kill. Moreover, we recognize that RBM does not provide the only indicator of physical fitness. Increased body mass does, however, correlate to decreased cardio-respiratory fitness [34], which is central to activities among hunter gatherers that use more basic killing technology in less developed regions of the world [35]. Additionally, presumably concerned with advertising the likelihood of securing large game to potential clients, guides might be more likely to post pictures of larger carcasses among potential photographs than unguided hunters, thus leading to a reporting bias. On the other hand, unguided hunters might also selectively post larger kills. If any bias exists, it might not only drive the strong guide effect we detected but also influence the detectability of other effects for which we tested. Without access to the pool of photographs that did not get posted by guided and unguided hunters, we cannot test for evidence of bias. We note, however, that our large sample set, which integrates data across species and populations over a vast region, provides more generalizable insight than a case study for which specific kill details were known.

Should the patterns we detected be representative, several implications emerge that relate not only to size-selection but also to wildlife hunting in general. Among them is that physical traits important among natural predators seem unimportant in the context of contemporary wildlife hunting. For example, despite the prevalence of camouflage among sampled hunters (i.e., 80%) and hunters in general (in 2011, 94% of North American hunters purchased at least one camouflage item [36]), this was the least important variable in predicting our measure of hunting performance. Although marketed to conceal hunters from visual detection by prey, at least two lines of evidence align with our results and suggest limited efficacy. First, Hall et al [37] recently showed with computer-based experiments that detection and capture of moving targets by humans does not vary with the presence or type of camouflage among targets; although to our knowledge not tested, we suspect that any crypsis benefits provided by camouflage clothing might likewise be compromised when hunters (commonly) move during hunts. More importantly, whereas vision serves as the dominant sense among humans who design and adopt camouflage clothing [38], most other mammals, cervids included, rely heavily on other sensory modalities to detect danger; namely, audition and olfaction [39]. This reality potentially further diminishes any benefit of camouflage clothing during hunting.

We also found evidence that physical fitness (as assessed by RBM) was unimportant in size-selective harvests or perhaps influential in an unexpected direction. Hunters with the highest RBM, presumably the most handicapped by adipose tissue, were no less likely to kill large prey. In fact, a modest interaction term suggested that, when unguided, larger hunters might actually have greater odds of killing large prey. We speculate that these patterns might relate to relationships among lifestyles, technology, and landscapes of hunters in the developed world. At the most basic level, even the most dedicated of hunters are nutritionally de-coupled from their prey; true subsistence hunting is rare [40]. Instead, hunters are subsidized considerably by commercially-supplied foods, which often underlie obesity [41]. Moreover, compared to other predator-prey interactions among vertebrates, bullets effectively obviate pursuit and capture of prey. Additionally, roads and vehicles decrease distance travelled on foot. In one study in Pennsylvania, USA, in which deer hunters were outfitted with telemetry equipment, movement averaged 5.5 km/day and only 0.8 km from roads [42]. Indeed, contemporary hunting is classified as a ‘light to moderate activity’ appropriate for all ages [43]. Finally, we speculate that the modest positive association we observed between RBM and prey size in unguided hunters might be mediated by socio-economic background. Specifically, people with low educational attainment hunt more [44]; generally, this same group also has a higher prevalence of obesity [45]. Accordingly, a relationship between higher RBM and larger prey might relate to increased experience (and knowledge) among those hunters.

We detected no patterns in hunting performance associated with age, which differs not only from natural predator systems (*see* Introduction) but also from hunter-gatherer systems. Among hunter-gatherers with limited technology, interactions among age, knowledge, and physical fitness are important [35], [46]–[48]. For example, Walker et al, (2002) found that among six hunter-gatherer societies, hunting return rates consistently peaked between the early 30 s and 50, well after peak physical fitness, and then declined thereafter with senescence. We failed to detect an association with age likely because of the inter-individual variation in both the expression of grey hair (our gross measure of age) and the age at which physical handicaps would counteract any benefit of accumulated knowledge. Additionally, as we explain above, in most cases hunting requires minimal physical exertion, which might permit men of a wide range of ages (and associated fitness) to perform equivalently.

Whereas efficient killing technology, vehicles, and road access have likely reduced the importance of physical ability, our finding that guides enable size-selective harvests suggests that knowledge might remain important. In effect, guides serve as ‘specialized predators’ that on average likely possess more intimate knowledge of localized hunting areas than unguided hunters. Indeed, in BC and Alberta, guides maintain defined territories in which knowledge would accumulate with experience. Moreover, there would be strong financial incentive for knowledge accumulation and subsequent sharing with clients. Finally, we acknowledge that if these territories are remote, guided hunters might be accessing different populations than unguided hunters. Prey in lightly-hunted guiding territories might be less alert and large specimens thus easier to kill. Additionally, higher proportions of larger individuals might be available to guided hunters in these areas. On the other hand, unguided hunters are legally entitled to use hunting grounds within guide territories. Detailed geographic information about the kills, however, was not available from our online data sources to examine whether guides accessed specific areas unguided hunters did not. We note, too, that these alternative explanations can complement and need not replace our knowledge-based interpretation of the effect of guides.

Wildlife hunters are unique and influential predators, so understanding the processes involved in hunting in general, and the mechanisms that enable size-selective harvests in particular, is important. To our knowledge, however, few others have posed questions like ours. Our preliminary and modest inquiry has revealed that, whereas the requirements to detect, pursue, and capture prey are common among all mammalian predators, the outcomes of predator-prey interactions between hunters and cervids in our systems and likely many others are governed more by knowledge than physical ability. In fact, contemporary technology and landscapes (i.e., “roadscapes”), themselves a feat of human intellect, likely compensate for the generally poor physical ability of hunters compared to carnivores. Guides, for whom we know of no analogue in other mammalian predator systems, seem particularly important in wildlife hunting. They serve as specialized knowledge holders and, among our samples, appear to enable size-selective harvests. Accordingly, should this association be real and widespread, wildlife managers interested in addressing the potential effects of selective harvests might benefit from a focus on guided hunting.

## Supporting Information

Dataset S1 (XLSX)

File S1 **Contains supporting tables.** Table S1. Full set of candidate models from which top model set emerged (main manuscript Results). Tables S2 & S3. Results from top model sets with Relative Body Mass (RBM) of hunters measured as a continuous variable. Patterns detected generally concur with models using small and large RBM categories, with all four parameters remaining the top model set. The main difference is that interaction term (guide x RBM) is no longer important.
(DOCX)
