# Supplementary material for: What Enables Size-Selective Trophy Hunting of Wildlife?
Source: PLoS One. 2014 Aug 6;9(8):e103487. doi: 10.1371/journal.pone.0103487 (PMC4123890; doi:10.1371/journal.pone.0103487)
Supplement: File S1 — Contains supporting tables. Table S1. Full set of candidate models from which top model set emerged (main manuscript Results). Tables S2 & S3. Results from top model sets with Relative Body Mass (RBM) of hunters measured as a continuous variable. Patterns detected generally concur with models using small and large RBM categories, with all four parameters remaining the top model set. The main difference is that interaction term (guide x RBM) is no longer important. (DOCX) [file pone.0103487.s002.docx]

**S2**

**What enables size-selective trophy hunting of wildlife?**

**Table S1.** Full set of candidate generalized linear models to predict the odds of wildlife hunters killing large cervids in British Columbia and Alberta, Canada.

| **Model Form** | ∆**AIC** | **ω_i_** |
| --- | --- | --- |
| guide | 0.0 | 0.22 |
| guide, RBM, guide*RBM | 0.3 | 0.19 |
| guide, age | 0.5 | 0.17 |
| guide, RBM | 1.3 | 0.12 |
| guide, camo | 1.9 | 0.09 |
| guide, RBM, age | 1.9 | 0.09 |
| guide, age, guide*age | 2.4 | 0.07 |
| guide, camo, age | 2.5 | 0.06 |
| age, RBM | 62.0 | <0.01 |
| age, camo | 62.6 | <0.01 |
| age, RBM, age*RBM | 63.2 | <0.01 |
| RBM | 64.8 | <0.01 |
| camo | 65.9 | <0.01 |

**Table S2.** Top models (∆AIC≤2) to predict the odds of wildlife hunters killing large cervids in British Columbia and Alberta, Canada.

| **Model Form** | ∆**AIC** | **ω_i_** |
| --- | --- | --- |
| guide | 0.0 | 0.26 |
| guide, age | 0.5 | 0.21 |
| guide, RBM | 1.9 | 0.10 |
| guide, camo | 1.9 | 0.10 |
| guide, RBM, guide*RBM | 2.0 | 0.10 |

**Table S3.** Model-averaged parameter estimates and relative importance derived from inference across the top model set.

| **Variable** | **Estimate** | | **P** | **Odds Ratio** | **95% CI** | ∑ **AIC ω_i_** |
| --- | --- | --- | --- | --- | --- | --- |
| guide | | 1.21 | **0.01** | 3.36 | 1.41-7.99 | 1.00 |
| age | | 0.10 | 0.23 | 1.10 | 0.94-1.29 | 0.33 |
| RBM | | 0.05 | 0.54 | 1.06 | 0.89-1.25 | 0.33 |
| RBM*guide | | -0.14 | 0.17 | 0.87 | 0.71-1.06 | 0.12 |
| camo | | 0.02 | 0.80 | 1.03 | 0.84-1.25 | 0.12 |
